# Construction of Green System for Flavonoids from *Dalbergia Pinnata* (Lour.) Prain Based on NADES-UAE: Intelligent Optimization–Molecular Mechanism–Activity Verification

**DOI:** 10.3390/ijms27104268

**Published:** 2026-05-11

**Authors:** Haiyu Yang, Bingyou Luo, Jingmin Mo, Junhui Xie, Jianwei Luo, Kunying Yu, Jianhua Wei, Haiyi Zhong

**Affiliations:** College of Pharmacy, Guangxi University of Chinese Medicine, Nanning 530200, China

**Keywords:** natural deep eutectic solvent, machine learning, extraction mechanism, *Dalbergia pinnata* (Lour.) prain, flavonoids

## Abstract

To promote green chemistry and improve the utilization of plant resources, flavonoids from *Dalbergia pinnata* (Lour.) Prain were extracted in this study by combining NADES (natural deep eutectic solvents) with UAE (ultrasound-assisted extraction). Among the 13 synthesized NADES, choline chloride (ChCl)–urea (NADES-13) exhibited the highest extraction rate, outperforming traditional organic solvents. The optimal conditions determined by response surface methodology (RSM) were as follows: ChCl–urea molar ratio of 1:3, moisture content of 60%, liquid-to-material ratio of 28.5 mL/g, ultrasonic extraction time of 49 min, and temperature of 62 °C. Under these conditions, the extraction rate reached 117.95 ± 5.97 mg/g, a 73.5% improvement compared with 80% EtOH extraction. The comparison of the two algorithms showed that RSM (R = 0.9981, RMSE = 0.6570) had better fitting accuracy and prediction stability under small sample conditions than MLP (R = 0.9427, RMSE = 5.261) and RF (R = 0.9431, RMSE = 5.2442). DFT (density functional theory) analysis demonstrated that hydrogen bonds, Van der Waals forces, and cation–π interactions mediate the interaction between NADES-13 and flavonoids. Ultrasonic cavitation-induced cell wall damage and the hydrogen-bond network of NADES-13 were confirmed separately by SEM (scanning electron microscopy) and FTIR (Fourier transform infrared spectroscopy). In vitro experiments showed that the extract possessed concentration-dependent antioxidant activity and strong antibacterial activity, with an inhibition rate of 96.87 ± 5.09% against *Escherichia coli* at a concentration of 0.04 mg/mL. In this study, a “Smart Optimization–Molecular Mechanism–Activity Verification” green extraction system was developed, which offers an efficient and environmentally friendly strategy for extracting plant bioactive components and contributes to the progress of green chemistry.

## 1. Introduction

*Dalbergia pinnata* (Lour.) Prain (*D. pinnata*) belongs to the genus *Dalbergia* of the Fabaceae family and is a highly valuable hardwood germplasm resource in China, with a natural distribution across Hainan, Guangxi, Yunnan, and other provinces [1]. This species is valuable not only as a high-quality hardwood resource but also for its medicinal applications, which have been widely documented [2]. In the traditional medical system, *D. pinnata* is a commonly used medicinal material for the treatment of rheumatic arthralgia, traumatic contusions, and tendon sprains, exerting core effects of reducing swelling and dissipating blood stasis, as well as dredging collaterals to alleviate pain [3]. With advances in modern pharmacological research, the medicinal value of *D. pinnata* has been further elucidated. Numerous relevant investigations have confirmed that it has multiple pharmacological activities, encompassing antibacterial, antioxidant, anti-inflammatory, antidiarrheal, cognitive-improving, anxiolytic, and tyrosinase inhibitory activities. These biological activities are mainly attributed to flavonoids, coumarins, and triterpenoids present in the plant. Among the above components, flavonoids have become a research focus in natural product research due to their multiple bioactivities, such as antibacterial, antioxidant, and neuroprotective properties, exhibiting outstanding application potential in developing new natural antibacterial preparations and auxiliary conditioning products for neurodegenerative disorders. *D. pinnata* is rich in flavonoids, which can not only significantly enhance the inhibitory effect on a variety of pathogenic bacteria but also effectively alleviate the oxidative stress response of the organism and regulate the metabolic balance of neurotransmitters in the brain, thus playing a key role in improving cognitive dysfunction and delaying the progression of neurodegenerative lesions of neural tissues [4]. Given the high application potential of flavonoids from *D. pinnata*, the development of a green and efficient extraction process is of practical significance for further investigation of their biological activities and potential applications in the pharmaceutical and health fields [5].

In the past few years, organic solvents have been the dominant medium for extracting bioactive constituents from medicinal herbs. Although conventional organic solvents can extract bioactive constituents effectively, they are often toxic and volatile, posing risks to both the environment and human health. The extraction approach relying on conventional organic solvents is inconsistent with the core tenets of toxicity reduction and environmental protection advocated by green chemistry [6]. Hence, the development of environmentally friendly extraction solvents for isolating and extracting natural bioactive components from medicinal herbs has emerged as a key research direction for promoting the advancement of green chemistry [7]. The advent of natural deep eutectic solvents (NADES) has brought a promising alternative to this research field, and their intrinsic properties are highly consistent with the core requirements of the 12 Principles of Green Chemistry proposed by the American Chemical Society (ACS), including the adoption of safer solvents, the design of degradable chemical substances, the reduction of waste generation, and the improvement of energy use efficiency [8]. NADES [9] are liquid systems formed by the association of two or more distinct components through hydrogen-bonding interactions, featuring a low melting point and the ability to achieve efficient dissolution of a wide range of polar and non-polar compounds [10]. Unlike conventional organic solvents, NADES possess lower toxicity and superior biodegradable properties, making them able to greatly reduce the harmful impacts on the ecological environment and human health. Furthermore, their composition and physicochemical properties can be flexibly regulated according to practical extraction requirements, thereby enabling the highly selective and soluble extraction of target components. In addition, NADES demonstrate better stability and recyclability during extraction operations, which can effectively reduce energy consumption and solvent loss in the extraction process. Benefiting from these prominent characteristics, NADES serve as an ideal substitute for traditional organic solvents and have been extensively utilized in research related to the extraction of naturally occurring bioactive components from plants—including phenolics, alkaloids, flavonoids, saccharides, and diterpenoids [11]. Numerous studies have confirmed that NADES possess significantly higher extraction efficiency than conventional organic solvents. This finding not only verifies the practical feasibility of NADES as a substitute for traditional extraction solvents but also highlights their important application value in the development of green chemistry.

In the advancement of green chemistry, the innovative application of green solvents and the development and promotion of energy-efficient extraction technologies must be advanced in tandem, a development concept that is highly aligned with the core tenets of United Nations Sustainable Development Goal 7: Affordable and Clean Energy and confirms the profound intrinsic link between green chemistry and the global sustainable energy strategy [12,13]. However, traditional extraction methods for plant bioactive components like maceration and reflux extraction are commonly plagued by low extraction efficiency, prolonged processing durations, and poor selectivity toward target components, making them unsuitable for meeting the dual requirements of environmental sustainability and extraction efficiency [14]. Thus, integrating green solvents with advanced high-efficiency extraction technologies has become a key approach to overcoming the technical bottlenecks of traditional extraction processes. Ultrasound-assisted extraction (UAE) exhibits excellent compatibility with various green solvents and demonstrates remarkable technical advantages in the extraction of plant bioactive components [15]. Its core mechanism of action stems from the cavitation effect induced by ultrasonic waves, which can effectively disrupt the plant cell wall structure and facilitate the release and dissolution of intracellular bioactive components into the solvent. At the same time, strong turbulence generated by ultrasonic vibrations can notably reduce the thickness of the mass transfer diffusion boundary layer and speed up the mass and heat transfer of the extraction system. It not only markedly elevates the extraction efficiency of target constituents but also largely shortens the extraction duration and decreases energy consumption, thereby enhancing resource utilization efficiency and the overall sustainability of the extraction procedure [16]. For this reason, the combination of this technology with green solvents yields a significant synergistic effect, driving the evolution of extraction processes toward greater efficiency, low carbon emissions, and environmental friendliness, and is highly consistent with the core development requirements of green chemistry [17]. As a typical representative of green solvents, NADES form the NADES-UAE method when combined with UAE technology, which exhibits excellent application potential in the extraction of plant bioactive components [18]. Relevant studies have applied this method to the extraction of flavonoids from *Sophorae Tonkinensis Radix et Rhizoma*, and after optimization of process conditions, the flavonoid extraction rate reached 3.785 ± 0.026 mg/g, a value significantly superior to that obtained via the conventional EtOH extraction method, which fully validates the high efficiency of this integrated technology [18]. Overall, the NADES-UAE method integrates high extraction efficiency and environmental friendliness and is a highly valuable green extraction technology for plant bioactive components [19].

**Figure 1:**
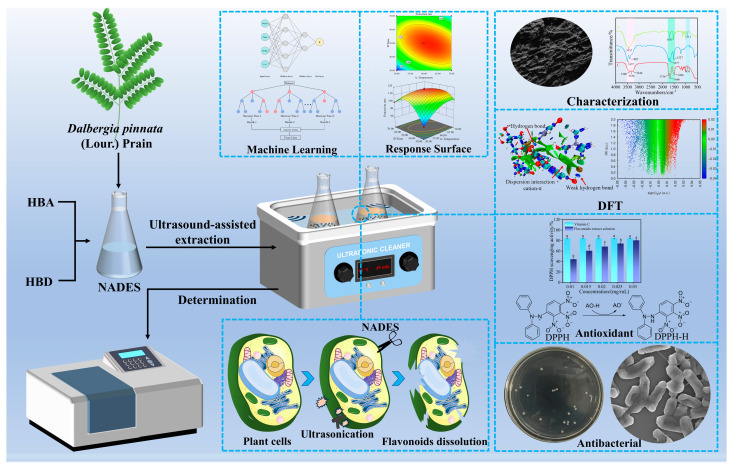
Schematic diagram of process optimization, characterization, mechanism, and activity evaluation for flavonoid extraction from *D. pinnata* using NADES via UAE.

While the NADES-UAE technique has been effectively applied to extracting flavonoids from diverse plant varieties, no reports have been published regarding its use in flavonoid extraction from *D. pinnata* up to now. Addressing the industry challenges associated with flavonoid extraction from *D. pinnata,* including low efficiency, environmental concerns, and the lack of molecular-level verification of the extraction mechanism, this study constructs a green extraction system of “Smart Optimization–Molecular Mechanism–Activity Verification”. Using green NADES as the solvent combined with UAE technology, precise process optimization was achieved through a dual algorithm of response surface methodology (RSM) and Machine Learning. Density functional theory (DFT) was adopted to disclose the extraction mechanism from a quantum chemical perspective, and the high biological activity of the extract was finally confirmed through relevant experiments (Figure 1). This provides an integrated solution for the green development of *D. pinnata*, a valuable resource.

## 2. Results and Analysis

### 2.1. Synthesis and Formation Mechanism of Nades

**Figure 2:**
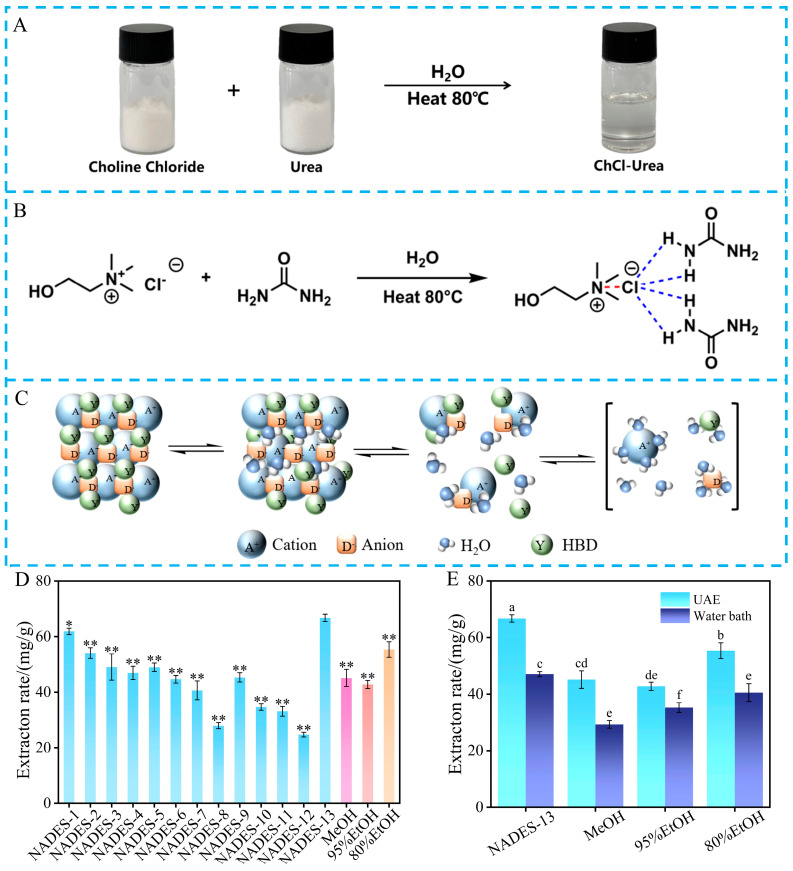
(**A**) Preparation process of NADES-13; (**B**) schematic diagram of hydrogen-bonding interactions; (**C**) structural formation model; (**D**) extraction rates of different solvents. * and ** indicate significant differences compared with NADES-13 (*p* < 0.05 and *p* < 0.01, respectively); (**E**) comparison between UAE and water bath extraction.

The 13 NADES prepared herein were all clear and transparent liquids with colors ranging from colorless to pale yellow. These solutions possessed excellent solubility, homogeneity, and stability, and no distinct irregularities were found in the corresponding systems. Taking ChCl–urea as an example, Figure 2A–C show its synthesis process, hydrogen-bonding interactions, and structural formation mechanism. As a hydrogen bond acceptor, ChCl provides Cl^−^, and urea acts as a hydrogen bond donor containing -NH_2_ moieties. Upon heating at 80 °C with a small amount of water, stable hydrogen bonding occurs between Cl^−^ and -NH_2_, leading to the formation of a homogeneous and clear NADES from the two solid starting materials. In pure NADES, the cations of choline chloride, Cl^−^, and urea molecules are closely assembled via intensive hydrogen bonds, forming a stable microstructure. With increasing water content, water molecules gradually fill the gaps between aggregates, leading to the breakage of hydrogen bonds, structural loosening, and eventual dissociation of each component into independent ions or molecules. By altering the stability of the hydrogen-bonding network, water content brings about significant changes in physical characteristics of NADES such as viscosity and electrical conductivity, thus realizing efficient control over the overall condition of the system [8].

### 2.2. Selection of the Optimal Nades and Extraction Method

As shown in Figure 2D, there were certain differences in the extraction rate of flavonoids from *D. pinnata* among 13 different NADES, with the extraction rate ranging from 24.71 ± 0.83 mg/g to 66.77 ± 1.3 mg/g. Such variations in extraction rate are thought to be strongly associated with the distinct physicochemical properties of different NADES [20]. Among all tested NADES, NADES-13 displayed the best extraction performance, with an extraction rate of 66.77 ± 1.3 mg/g, representing a 170.2% increase compared with NADES-12. In contrast to conventional extraction solvents, NADES-13 achieved a markedly higher extraction rate than 80% EtOH (55.36 ± 2.8 mg/g) and other traditional solvents, with the advantages of higher extraction efficiency and lower solvent usage, which accords with the core concept of green chemistry. Meanwhile, previous studies have confirmed that choline chloride–urea-based NADES can be regenerated and reused without a significant loss of extraction efficiency [21], further expanding the application potential of such systems in sustainable extraction. To investigate the influence of ultrasonic treatment on flavonoid extraction, the extraction behaviors of water bath extraction and UAE were compared in this work, and the corresponding results are presented in Figure 2E. When NADES-13 was applied as the extraction medium, the extraction rate of UAE was 40% higher than that of water bath extraction. Even when traditional organic solvents were employed for flavonoid extraction, UAE still exhibited more favorable extraction efficiency. These results indicate that UAE improved the extraction efficiency, especially when combined with the NADES system, making it a promising method for the eco-friendly and high-efficiency extraction of flavonoids from *D. pinnata*.

### 2.3. Analysis of the Single-Factor Experiment

**Figure 3:**
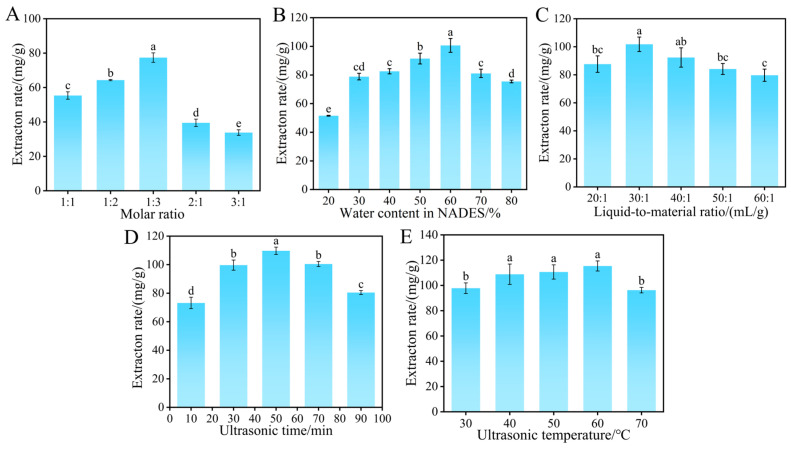
(**A**–**E**) Effects of molar ratio, water content, liquid-to-material ratio, ultrasonic time, and temperature on the extraction rate.

In the present work, the influence of the molar ratio between ChCl and urea on flavonoid extraction rate was initially investigated (Figure 3A). The results revealed that the extraction performance reached its maximum at a molar ratio of 1:3, with the corresponding extraction rate being 77.40 ± 2.75 mg/g. This ratio could effectively improve the transfer efficiency of flavonoids and enhance the stability of the extraction system [22]. When the molar ratios were 1:4 and 1:5, crystallization occurred in the extraction system, which was unfavorable for flavonoid extraction. Therefore, these two ratios were excluded, and 1:3 was ultimately determined as the optimal molar ratio for subsequent experiments.

Figure 3B illustrates the effect of water content on the extraction rate of flavonoids. Within the water content range of 20% to 60%, the flavonoid extraction rate increased gradually with rising water content and reached a maximum value of 100.66 ± 4.8 mg/g at 60% water content. A further increase in water content resulted in a declining trend of the extraction rate. A suitable water content can lower the viscosity of NADES and facilitate the release of flavonoids. Nevertheless, excessive water content alters the solvent polarity and disrupts the hydrogen-bond network in the system, thereby reducing the extraction efficiency [23]. Accordingly, a water content of 60% was chosen for the following experiments.

The influence of various liquid-to-material ratios on the flavonoid extraction rate from *D. pinnata* is displayed in Figure 3C. As the liquid-to-material ratio increased, the flavonoid extraction rate presented a tendency to rise first and then decline, attaining the maximum value of 101.79 ± 5.16 mg/g at a ratio of 30:1 (mL:g). A proper increase in solvent volume can enlarge the contact interface between the extraction solvent and the raw material, thus accelerating the dissolution of flavonoids. Nevertheless, an excessively high liquid-to-material ratio may lead to the dispersion of ultrasonic energy, and the release of impurities can also interfere with the extraction process [24]. Consequently, 30:1 (mL:g) was selected as the optimal liquid-to-material ratio.

The influence of ultrasonic time on the flavonoid extraction rate is illustrated in Figure 3D. As the ultrasonic treatment time was prolonged, the flavonoid extraction rate increased first and then decreased, reaching a maximum of 109.72 ± 2.59 mg/g at 50 min. This may be because most flavonoids had already been extracted after 50 min; further extending the treatment time would not only increase the dissolution of impurities but might also lead to flavonoid degradation [25]. Therefore, the optimal ultrasonic time was identified as 50 min.

Ultrasonic temperature was also a crucial factor influencing the flavonoid extraction rate. As indicated in Figure 3E, with the increase of ultrasonic temperature, the extraction rate first rose and then declined, reaching a maximum value of 117.78 ± 4.34 mg/g at 60 °C. Properly increasing the temperature could accelerate the molecular movement rate, thereby enhancing the efficiency of flavonoid extraction; however, when the temperature exceeded 60 °C, the stability of hydrogen bonds in the system would decrease, leading to the degradation of flavonoids [26]. For this reason, 60 °C was chosen as the optimal ultrasonic temperature.

### 2.4. Machine Learning Results

#### 2.4.1. Artificial Neural Network Model

To obtain the minimum root-mean-square error (RMSE) and maximum correlation coefficient (R) while avoiding model overfitting, this study selected a double hidden layer structure (5, 2) with 1500 training epochs through multiple experiments to conduct nonlinear regression analysis of the flavonoid extraction process. This double hidden layer structure effectively balanced the model complexity and prediction accuracy (excessively complex structures are prone to overfitting), and the specific performance indicators of the model at this point are R = 0.9427, RMSE = 5.261.

Based on the extended form of the Garson algorithm [27], this study analyzed the contribution degree of each input variable to the flavonoid extraction rate. By computing the sum of absolute values of the weight matrix from the input layer to the hidden layer (Table 1), the results demonstrated that water content and ultrasonic time represented the core factors influencing the extraction rate, followed by ultrasonic temperature, whereas the liquid-to-material ratio exerted the weakest effect on the extraction rate. To validate the reliability of the analytical outcomes, feature ablation experiments were further carried out in this work. Through eliminating each process parameter one by one and monitoring the variations in the model’s R and RMSE values, the experimental findings were in accordance with the analytical conclusions of the Garson algorithm (Table 2).

With the liquid-to-material ratio at 30:1 (mL:g), this study conducted a simulation analysis of the nonlinear relationship between water content, ultrasonic time, ultrasonic temperature, and flavonoid extraction rate. The optimal process parameters obtained by simulation were ultrasonic temperature 50 °C, ultrasonic time 62 min, water content 47%, with a corresponding predicted extraction rate of 119.97 mg/g; the extraction rate obtained by actual experiments was 112.26 ± 0.46 mg/g, and the predicted and experimental values showed good agreement.

#### 2.4.2. Random Forest Model

Random Forest performs predictive analysis by aggregating multiple decision trees, utilizing the randomness of data to mitigate overfitting, and is particularly suitable for research scenarios with small sample sizes and multiple features. The model performance was verified through 10-fold cross-validation, and the results showed that the model has high fitting accuracy (R = 0.9431, RMSE = 5.2442); its performance is close to that of the Artificial Neural Network (R = 0.9427, RMSE = 5.261). Although these two prediction methods are based on different theoretical foundations, their prediction results were in good agreement, and both successfully captured the major trends in the flavonoid extraction process.

The outcomes of the feature ablation experiment revealed that the importance order of each input feature was in line with the analytical results of the Artificial Neural Network. With the liquid-to-material ratio fixed at 30:1 (mL:g), the optimal process parameters were acquired via simulation analysis: ultrasonic temperature of 55 °C, ultrasonic time of 52 min, and water content of 51%, with a corresponding predicted extraction rate of 117.46 mg/g; the extraction rate obtained from actual verification experiments was 111.65 ± 2.22 mg/g, suggesting a satisfactory consistency between the predicted and measured values.

### 2.5. Response Surface Analysis

#### 2.5.1. Regression Model Fitting and Analysis of Variance

The regression model developed in this work adopted ultrasonic temperature, ultrasonic time, and liquid-to-material ratio as input variables (marked as A, B, and C, respectively), with the extraction rate as the response variable. A stepwise regression method was used to eliminate non-significant terms, resulting in the standard second-order regression model (Equation (1)):

(1) $$\begin{array}{l} \mathrm{Extraction}\;\mathrm{rate}\;(\mathrm{mg}/g)\;=\;116.93\;+\;2.94A\;+\;0.5762B\;-\;1.72C\;-\;8.25\mathrm{AB}\;+\\ 0.2321\mathrm{AC}\;+\;2.35\mathrm{BC}\;-\;7.20A^{2}\;-\;15.9B^{2}\;-\;5.95C^{2} \end{array}$$

**Figure 4:**
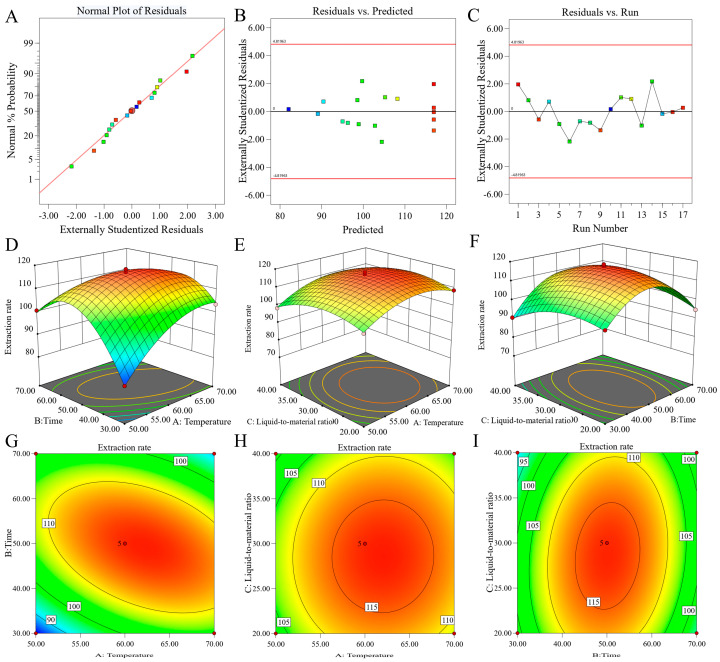
(**A**) Normal probability plot of residuals; (**B**) residuals vs. predicted values; (**C**) residuals vs. run order; (**D**–**F**) 3D response surface plots; (**G**–**I**) contour plots.

Table 3 lists the analysis of variance results corresponding to the extraction rate data. A model can be regarded as effective when its *p*-value is below 0.05. The standard quadratic regression model obtained an F-value of 206.51, accompanied by a *p*-value of less than 0.0001. Meanwhile, the coefficient of determination exceeded 0.9, which reflected high consistency between the predicted data and the actual measured results and demonstrated excellent fitting precision of the model. This model was suitable for subsequent prediction and optimization of the extraction rate. Ultrasonic temperature had the highest F-value, indicating its greatest impact on the extraction rate. The sequence of influence strength was as follows: ultrasonic temperature > liquid-to-material ratio > ultrasonic time. Among all interaction effects, the interaction between ultrasonic temperature and ultrasonic time exerted the most notable influence on the extraction rate, whereas the interaction between ultrasonic temperature and liquid-to-material ratio had no remarkable impact. The model’s reliability was confirmed by means of residual analysis [28]. Figure 4A–C depict the fitting effect of the regression model on the flavonoid extraction rate of *D. pinnata*: Figure 4A shows the normal probability plot of residuals, in which the residual points are distributed along a straight line, suggesting that the residuals conformed to an approximate normal distribution; Figure 4B presents the plot of residuals versus predicted values, where the residual points are randomly scattered above and below the zero line without any outliers or abnormal points; Figure 4C illustrates the correlation between test sequence and residuals, with the residual points randomly distributed around the zero line and no obvious correlation observed. Analysis of variance verified the credibility of the constructed model, and the non-significant lack of fit demonstrated that the quadratic regression model can be reliably applied to predict the flavonoids extraction rate from *D. pinnata*.

#### 2.5.2. Analysis of Factor Interactions

To explore the influence of interactive effects on the extraction rate, three-dimensional response surface diagrams and contour plots were analyzed in detail [29]. When the liquid-to-material ratio was maintained at a medium level, Figure 4D,G illustrate the interaction between ultrasonic temperature and ultrasonic time. The interaction surface exhibited a steep slope, which indicated that the mutual effect of ultrasonic temperature and ultrasonic time exerted the most significant impact on the extraction rate—consistent with the findings of the variance analysis. At low temperatures, the extraction rate first rose and then declined as the ultrasonic time extended; at higher temperatures, the extraction rate increased slowly initially and then dropped sharply after 42 min, demonstrating a notable interactive effect between these two factors. Figure 4E,H depict the interaction between ultrasonic temperature and the liquid-to-material ratio, with a relatively flat interaction surface, suggesting that the combined impact of these two factors was not significant. Meanwhile, Figure 4F,I show the interaction between ultrasonic time and the liquid-to-material ratio: as the ultrasonic time increased, the extraction rate first increased and then decreased, with relatively large fluctuations. This further confirms the existence of a significant interactive relationship between these two factors. In summary, the interactive effect between ultrasonic temperature and ultrasonic time had the most prominent influence on the extraction rate.

#### 2.5.3. Optimization of Process Parameters and Experimental Validation

The optimal extraction conditions were determined via a second-order polynomial equation: ultrasonic temperature of 62.31 °C, time of 48.932 min, and liquid-to-material ratio of 28.495 mL/g, with a predicted extraction rate of 117.39 mg/g. For actual operational convenience, the predicted optimal conditions were moderately adjusted to 62 °C, 49 min, and 28.5 mL/g. The measured extraction rate was 117.95 ± 5.97 mg/g, reaching 100.5% of the predicted value, which verified the reliability of the model and the rationality of the parameters. Under the same conditions, the extraction rate with 80% EtOH was 67.99 ± 2.3 mg/g, and the NADES extraction rate was 73.5% higher than that. This demonstrated that NADES not only significantly outperformed traditional solvents in extraction efficiency but also achieved a balance between high efficiency and low environmental impact due to its green characteristics, highlighting the “green and efficient” advantage.

### 2.6. Comparison of Different Algorithms

**Figure 5:**
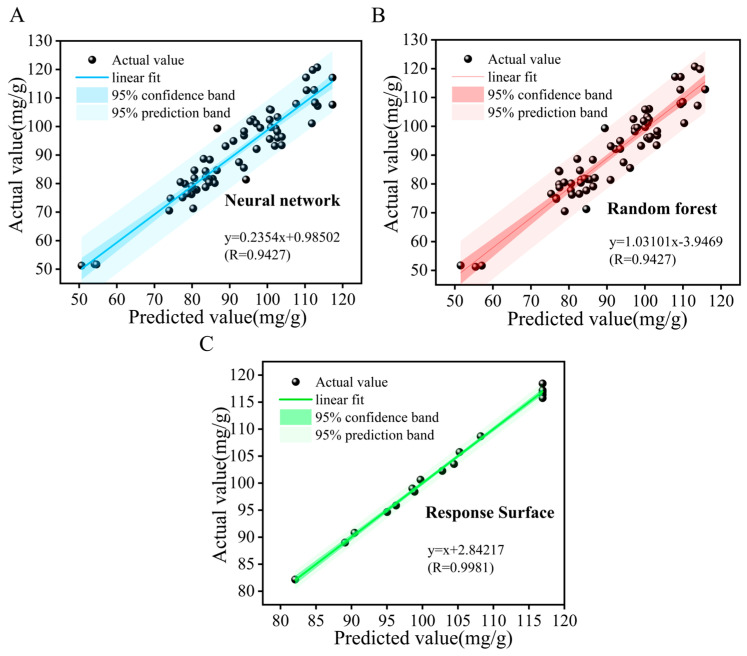
Comparison of predicted and actual values for the three models: (**A**) neural network, (**B**) random forest, and (**C**) response surface.

Table 4 compares the fitting and prediction performance of the three algorithm models, and Figure 5A–C display the deviation between the predicted and actual values of each model. In general, the closer the predicted values are to the measured values and the more concentrated the data points are distributed, the higher the prediction accuracy and reliability of the model. In Figure 5C, the data points of the RSM tightly cluster around the line of *y* = *x* + 2.84217, with almost no sample points showing significant deviation. The R reached as high as 0.9981, accompanied by an MAE of only 0.5373 and an RMSE of 0.6570, which were far lower than those of the neural network and Random Forest models. These results indicated that the RSM model not only achieved an extremely high degree of linear fitting for the experimental data but also yielded minimal deviation in a single prediction with stable and reliable outcomes. From the perspective of confidence intervals, the 95% confidence and prediction bands of RSM were extremely narrow and almost coincided with the fitting line. This demonstrated that within the parameter range of this study, the model maintained a stable prediction accuracy regardless of the low or high extraction rate interval, exhibiting excellent response sensitivity and consistency to the changes in process parameters. The predictions from the neural network (Figure 5A, R = 0.9427, MAE = 4.3552, RMSE = 5.261) and Random Forest (Figure 5B, R = 0.9431, MAE = 4.2762, RMSE = 5.2442) models showed some deviation. This could be attributed to the small dataset size and random errors. In small-sample cases, the response surface method was better at fitting the data, while the other models were more susceptible to noise. Although the results differed, they were generally consistent. In this study, the response surface model provided more accurate predictions and offered effective guidance from a green chemistry perspective to reduce solvent consumption and environmental impact.

### 2.7. DFT Analysis for Nades Extraction

**Figure 6:**
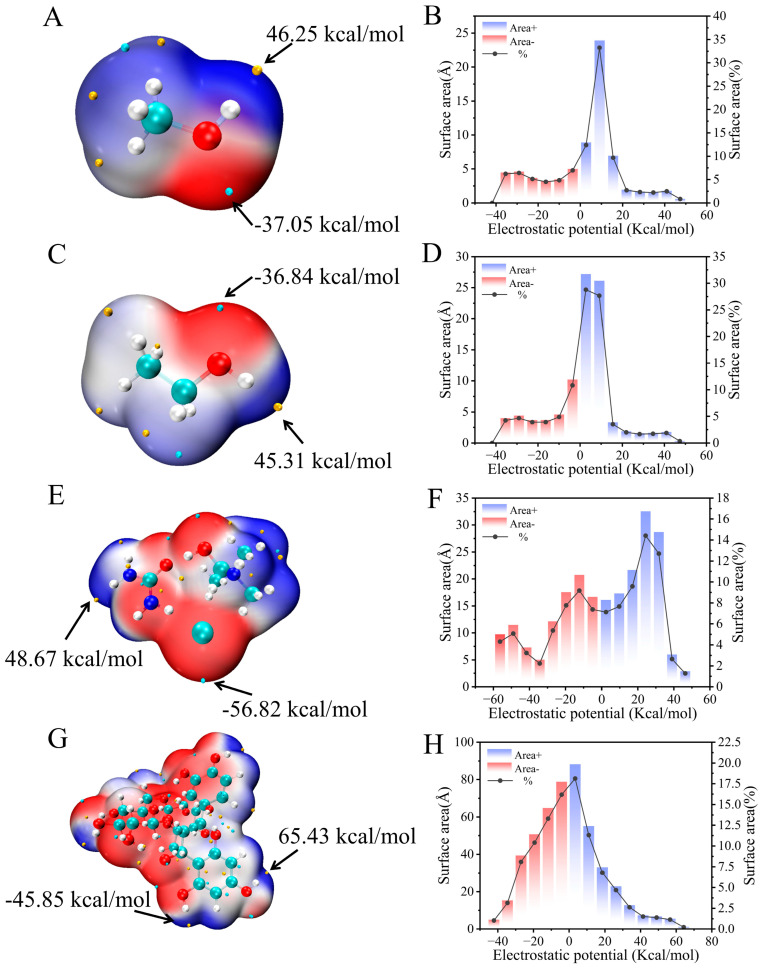
ESP mapping of the molecular Van der Waals surface and the surface area distribution of each ESP interval: MeOH (**A**,**B**), EtOH (**C**,**D**), NADES-13 (**E**,**F**), and rutin (**G**,**H**). The small yellow spheres represented the surface potential positive regions, while the small green spheres represented the surface potential negative regions.

Figure 6A,C,E,G show the ESP distributions of different solvents and rutin. Red regions stand for electron-rich sites, and blue regions correspond to electron-deficient sites, which is beneficial for analyzing the charge interactions between solvents and rutin [30]. The oxygen atoms in MeOH and EtOH form electron-rich regions around themselves, whereas the hydrogen atoms attached to oxygen form electron-deficient regions. These charge distributions enable MeOH and EtOH to form hydrogen bonds with the hydroxyl (-OH) groups of rutin, showing complementary electrophilic and nucleophilic properties. In NADES-13, the negative charge of ChCl (Cl^−^) creates a strong electron-rich region on the molecular surface, which corresponds to possible electrostatic interactions with rutin. The ESP distribution of rutin exhibits both electron-rich and electron-deficient regions around multiple hydroxyl groups, indicating that rutin displays both electrophilic and nucleophilic characteristics in its interactions with solvents. Quantitative analysis in Figure 6B,D,F,H further reveals the ESP differences between the solvents and rutin. The surface ESP of MeOH, EtOH, and NADES-13 is mainly distributed in positive charge regions, accounting for 66.95%, 67.61%, and 55.43%, respectively, while that of rutin is concentrated in negative charge regions with a proportion of 52.17%. These results illustrate that distinct interactions are established between solvent molecules and rutin through electrostatic attraction, and remarkable attraction occurs between the positively charged regions of solvents and the negatively charged regions of rutin, thus facilitating their interaction.

**Figure 7:**
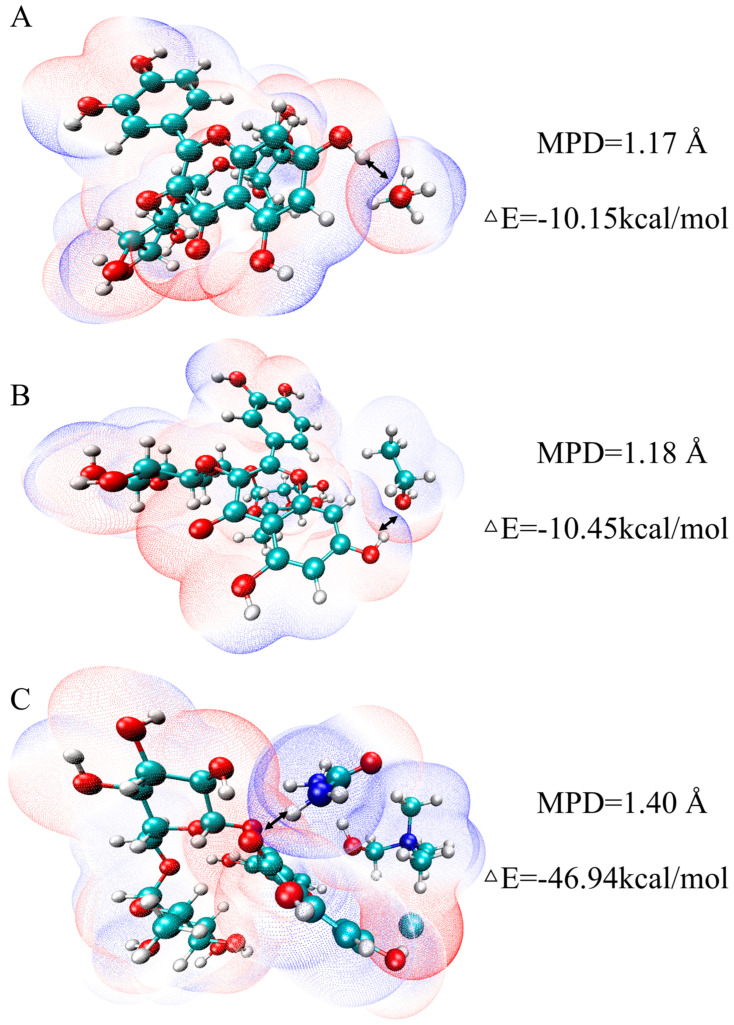
(**A**–**C**) Van der Waals penetration maps of MeOH, EtOH, and NADES-13 with rutin, respectively.

In terms of ΔE and mutual penetration distance (MPD) (Figure 7), the ΔE between NADES-13 and rutin was lower than −20 kcal/mol with an MPD of 1.4 Å, indicating extremely strong binding strength and favorable stability of the complex. Notably, this calculated value cannot represent the real interaction free energy, which could be determined by more accurate computational techniques [31]. The ΔE and MPD were −10.15 kcal/mol and 1.17 Å for MeOH with rutin, and −10.45 kcal/mol and 1.18 Å for EtOH with rutin, respectively. Compared with MeOH and EtOH, NADES-13 exhibited significantly stronger binding affinity and higher complex stability.

**Figure 8:**
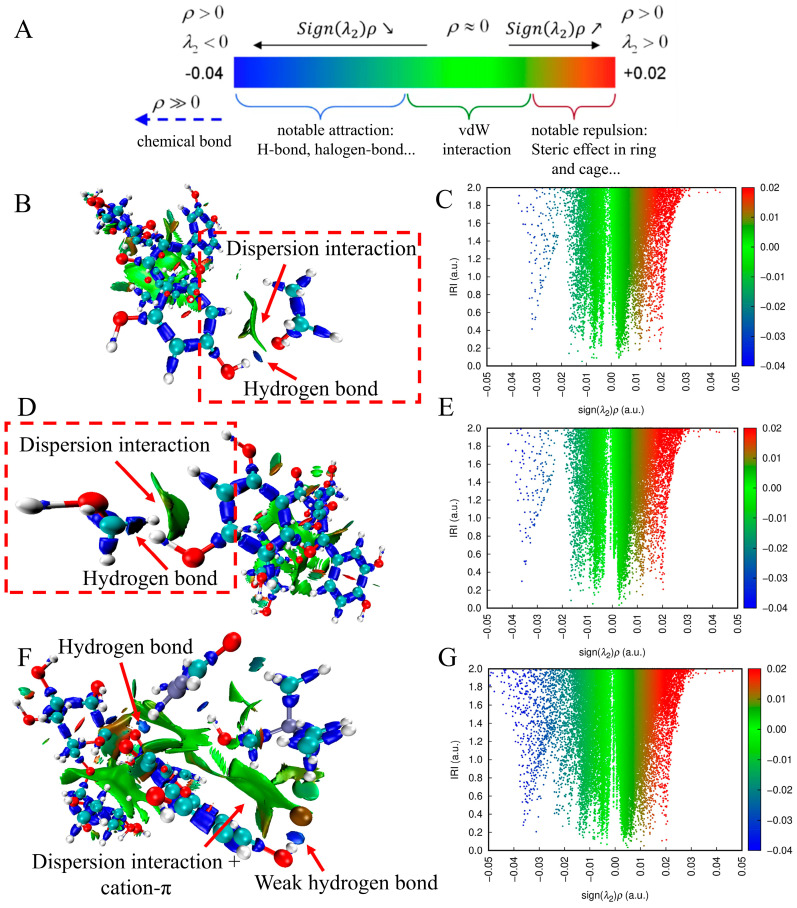
(**A**) Schematic diagram of the IRI equipotential surface color scale (colored by sign(λ_2_)ρ); (**B**,**C**) IRI equipotential surface map and scatter plot of MeOH and rutin; (**D**,**E**) IRI equipotential surface map and scatter plot of EtOH and rutin; (**F**,**G**) IRI equipotential surface map and scatter plot of NADES-13 and rutin.

Although ESP analysis preliminarily recognized the interaction regions between solvents and rutin, the specific binding sites and interaction intensities still need to be explored in detail. Therefore, this study further employed an independent gradient model (IRI) analysis for visualization and quantitative exploration. The color scale in Figure 8A clearly distinguishes the types of interactions: blue corresponds to strong attractive interactions, including hydrogen bonds, green stands for Van der Waals forces, and red signifies steric repulsive effects [32]. In the MeOH–rutin system (Figure 8B), the hydroxyl oxygen atom of methanol forms an O-H•••O hydrogen bond with the hydroxyl hydrogen atom on the benzene ring of rutin, and obvious Van der Waals interactions also exist between them. In addition, the α-C atom of methanol generates weak Van der Waals interactions with the π electrons of the rutin benzene ring. The interaction pattern between EtOH and rutin is analogous (Figure 8D), with the key distinction being that the β-C atom of ethanol takes part in the formation of Van der Waals interactions. The interaction network between NADES-13 and rutin is more intricate (Figure 8F): two kinds of hydrogen bonds (O-H•••O and O-H•••Cl) are formed between them, the N^+^ in NADES-13 generates cation–π interactions with the rutin benzene ring, and multiple Van der Waals forces are superimposed at the same time. The number of blue peaks with IRI values below 0.5 in its IRI scatter plot (Figure 8C,E,G) is also higher than that in the MeOH and EtOH systems, verifying a more abundant number of hydrogen bonds. The above results indicate that NADES-13 forms a stable supramolecular complex with rutin by constructing a denser network of weak interactions, which is the core molecular mechanism for its efficient extraction of rutin from *D. pinnata*, and also provides a theoretical basis for its application as a flavonoid extraction solvent.

### 2.8. Characterization Analysis

**Figure 9:**
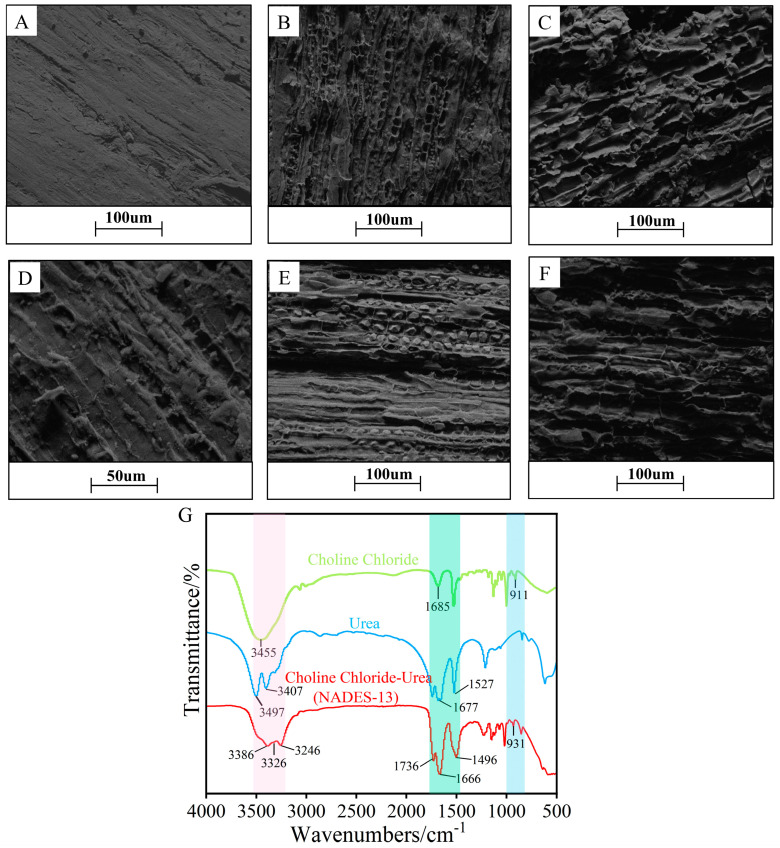
(**A**–**F**) Effects of different treatment methods on the surface structure of *D. pinnata*; (**G**) FTIR spectra of NADES and its components.

Figure 9A,D showed that the untreated *D. pinnata* powder had depressions and small protrusions on its surface at low magnification (×800), with a compact and smooth structure without visible cracks, displaying a natural texture. At higher magnification (×1600), cracks, irregular shapes, and small protrusions became visible, reflecting its physical properties and durability. In Figure 9B, the surface of the *D. pinnata* treated with NADES-13 exhibited roughness and unevenness, forming numerous loose, porous structures, which were effectively expanded and opened upon sufficient contact, facilitating the release of flavonoids. In contrast, Figure 9E showed that the *D. pinnata* treated with 80% EtOH had only a few pores opened, with the structure remaining closed, hindering extraction. Figure 9C depicts that ultrasonic-assisted NADES-13 treatment caused significant damage and cracks to the surface of *D. pinnata*, severely compromising the cell wall. Compared to the ultrasonic-assisted EtOH treatment in Figure 9F, the surface treated with NADES-13 was rougher, with deeper cracks and more prominent pores. This indicated that ultrasonic assistance could disrupt the dense fibrous structure, increase the contact area, and thereby promote the release of bioactive substances [33], and this pattern of action was consistent with the trend shown in the Graphical Abstract, where ultrasonic-assisted NADES was used for flavonoids extraction. Therefore, the combination of NADES-13 and ultrasonic assistance significantly enhanced the extraction rate by promoting flavonoids release, demonstrating a green and efficient extraction performance, which was superior to traditional EtOH extraction in both efficiency and environmental sustainability.

In this study, FTIR was employed to perform a comparative spectral analysis of the synthesized NADES-13 (ChCl–urea), as well as pure ChCl and pure urea. The results were shown in Figure 9G. Pure ChCl showed a characteristic absorption peak at 3455 cm^−1^, which was assigned to the hydrogen-bonded hydroxyl (H-O) group in the alcohol structure. On the other hand, NADES-13 showed a broad absorption band in the range of 3200–3600 cm^−1^, which was ascribed to the stretching vibrations of the O-H group in ChCl and the N-H group in urea [34], verifying the existence of hydrogen-bonding interactions inside NADES-13. Additionally, the characteristic absorption peaks of the C-N group at 911 cm^−1^ and the CH_2_ bending vibration at 1685 cm^−1^ observed in pure ChCl were also evident in NADES-13, albeit with slight shifts in wavenumber due to hydrogen bonding interactions. The FTIR spectrum of pure urea exhibited characteristic absorption peaks of N-H moieties at 3407 cm^−1^ and 3497 cm^−1^, corresponding to the asymmetric and symmetric stretching vibrations of N-H bonds, respectively. A distinct absorption peak at 1677 cm^−1^ verified the existence of the C=O group, and the signal at 1527 cm^−1^ reflected the stretching vibrations of the C-N group. The FTIR spectrum of NADES-13 demonstrated that the synthesized NADES retained the characteristic absorption peaks of the pure substances, with no new peaks emerging. This suggested that ChCl and urea successfully formed NADES-13 without generating new compounds, maintaining their original chemical structures. These results not only provided robust spectral evidence for the synthesis of NADES-13 but also further confirmed its synthesis mechanism.

### 2.9. Antioxidant Efficacy Analysis

**Figure 10:**
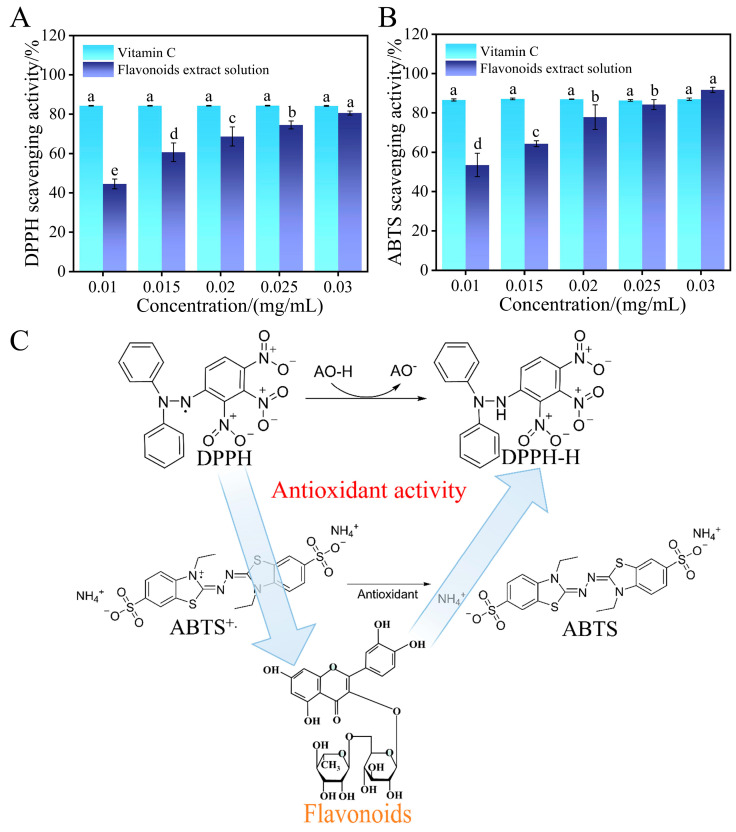
(**A**) DPPH· radical scavenging activity; (**B**) ABTS^+^· radical scavenging activity; (**C**) schematic diagram of the antioxidant mechanism of flavonoids.

In this study, DPPH· and ABTS^+^· assays were employed to evaluate the antioxidant activity of NADES-based flavonoid extracts at different concentrations. The results are shown in Figure 10A,B, and the reaction mechanism diagram (Figure 10C). In the DPPH· radical scavenging assay (Figure 10A), as the flavonoid concentration increased from 0.01 mg/mL to 0.03 mg/mL, the scavenging rate increased significantly from 44.54 ± 2.47% to 80.57 ± 1.06%, exhibiting a clear concentration-dependent pattern. This trend can be explained by the reaction mechanism in Figure 10C: the phenolic hydroxyl groups (-OH) in flavonoid molecules can donate hydrogen atoms to reduce DPPH· radicals to stable DPPH-H, thereby effectively neutralizing free radicals and alleviating cell damage caused by oxidative stress [35]. In the ABTS^+^· radical scavenging assay (Figure 10B), the scavenging rate of flavonoids increased from 53.56 ± 5.97% at low concentrations to 91.73 ± 1.32% at high concentrations, also showing a significant concentration dependence. As shown in Figure 10C, flavonoids reduce ABTS^+^· radicals to colorless ABTS by donating electrons. This process is similar to the DPPH· scavenging mechanism, but the more prominent increase in scavenging rate observed in the ABTS^+^· assay is related to the characteristics of ABTS^+^· radicals and their reaction mechanism with flavonoids, as ABTS^+^· radicals are more diffusible in aqueous solution. Compared with the positive control vitamin C, which maintained a relatively stable scavenging rate of approximately 84–86% in both assays, the antioxidant activity of flavonoids increased more significantly with concentration, and at higher concentrations, they exhibited stronger antioxidant activity, approaching or even exceeding that of vitamin C. This indicates that NADES-based flavonoid extracts can exert antioxidant effects through both hydrogen atom transfer and electron transfer mechanisms (Figure 10C), and also possess concentration-tunable antioxidant potential, providing broader prospects for their green applications in food, medicine, and other fields.

### 2.10. Antibacterial Performance Analysis

**Figure 11:**
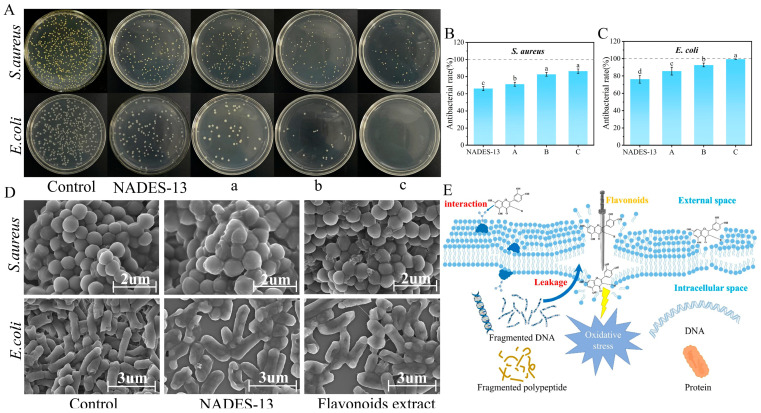
(**A**) Colony growth of the two bacterial strains on agar plates after incubation; (**B**,**C**) antibacterial rates of flavonoid extracts at different concentrations (0.013, 0.027, and 0.04 mg/mL, denoted as A, B, and C, respectively); (**D**) SEM of bacterial morphology; (**E**) schematic diagram of the proposed antibacterial mechanism of flavonoids.

This study assessed the antibacterial effects of the flavonoid extract against *S. aureus* and *E. coli* (Figure 11A) and performed a quantitative analysis of the antibacterial rates at varying concentrations (Figure 11B,C) [36]. Bacterial growth showed significant differences under different treatments: in the control group, both strains grew normally, indicating suitable culture conditions; after treatment with NADES-13, the growth of both bacteria was inhibited to a certain extent. The flavonoid extract exhibited particularly significant inhibitory effects on *E. coli*, and the antibacterial activity increased with increasing concentration. At the highest concentration, almost no visible colonies were observed, with an inhibition rate of 96.87 ± 5.09%. In contrast, *S. aureus* was less sensitive to the extract; even at the highest concentration, the inhibition rate was only 86.45 ± 2.48%, failing to achieve complete suppression. This difference in antibacterial effects may be related to the structural and compositional differences in the cell walls of the two bacteria [37,38].

To further reveal the antibacterial mechanism of the flavonoid extract and its correlation with bacterial species, this study used SEM to observe morphological changes in bacteria before and after treatment (Figure 11D) [39]. Untreated *E. coli* exhibited a typical rod shape, with tightly arranged cells and clear, intact boundaries. After treatment with NADES-13 and the flavonoid extract, most *E. coli* cells showed morphological abnormalities such as swelling and distortion, with depressions and vacuolization on the cell wall surface, indicating damage to the outer structure. In the control group, *S. aureus* appeared as regular spheres or ovals with a smooth and compact surface. After treatment, the cell wall of *S. aureus* showed obvious shrinkage and deformation, with impaired structural integrity. These observations suggested that the flavonoid extract exerted antibacterial effects, possibly by disrupting the integrity of the bacterial cell wall, thereby affecting the stability of the cell membrane and intracellular structures [40]. Notably, some *S. aureus* cells remained relatively intact under SEM, which is consistent with the quantitative results showing a lower inhibition rate and incomplete suppression (Figure 11A,B).

Combined with the schematic diagram of the antibacterial mechanism in Figure 11E, a more in-depth interpretation of the above phenomena can be made: flavonoids can interact with the bacterial cell membrane, destroy its integrity, and cause the leakage of key intracellular substances (e.g., DNA, polypeptides). At the same time, flavonoids can induce intracellular oxidative stress, further exacerbating cellular damage, ultimately inhibiting bacterial growth and even leading to cell death. Previous phytochemical studies on *D. pinnata* have reported the presence of multiple flavonoids, including pinocembrin, biochanin A, daidzein, luteolin, and quercetin [41]. Therefore, the antibacterial activity observed in the present study is likely associated with the combined action of multiple flavonoid constituents rather than a single monomeric compound. Nevertheless, since the detailed composition of the optimized NADES extract was not further characterized in this work, the present results should be interpreted as the overall antibacterial effect of a flavonoid-enriched fraction. Future studies should combine HPLC-MS/MS or bioactivity-guided analysis to clarify the major active constituents and their respective contributions to the antibacterial activity. Overall, the flavonoid extract exhibited strong antibacterial activity against both *E. coli* and *S. aureus*, with a more pronounced effect on *E. coli* under the tested conditions. These results suggest that the optimized NADES-derived flavonoid extract has promising antibacterial potential against these two representative bacterial strains [42].

## 3. Materials and Methods

### 3.1. Herb Acquisition and Reagents

The medicinal plant *D. pinnata* was sourced from the authentic herbal market in Wuming District, Nanning City, Guangxi Zhuang Autonomous Region. Choline chloride (ChCl), urea, and other NADES, as well as 2,2′-azino-bis(3-ethylbenzothiazoline-6-sulfonic acid) diammonium salt (ABTS^+^·) and rutin, were all purchased from Shanghai Aladdin Biochemical Technology Co., Ltd. (Shanghai, China) Methanol (MeOH) and ethanol (EtOH) were obtained from Chengdu Kelong Chemical Co., Ltd. (Chengdu, China).

### 3.2. Preparation of Nades

In this research, ChCl was mixed with diverse hydrogen bond donors (HBDs) according to a predetermined molar ratio, followed by magnetic stirring at 80 °C until a uniform, transparent solution was formed [43]. A total of 13 NADES prepared in the experiment were listed in Table 5.

### 3.3. Extraction and Quantification of Flavonoids

Exactly 0.5 g of *D. pinnata* crude powder was weighed and mixed with NADES at a liquid-to-material ratio of 30:1 (mL:g). The NADES employed was prepared at a molar ratio of 1:2 with a 30% water content [44]. An ultrasonic cleaner was used to treat the mixture via UAE for 30 min under the conditions of 360 W and 40 °C. After the extraction process, the mixture was centrifuged at 8000 rpm for 10 min, and the supernatant was collected as the flavonoid extract. Subsequently, 0.5 mL of the extract was transferred into a 25 mL volumetric flask. The flavonoid content was measured by the sodium nitrite–aluminum nitrate colorimetric method, and the extraction rate was calculated with reference to Equation (2) [45].

(2) Extraction rate (mg/g)= (C × 25 × n) / M × 100%

*C* represents the flavonoid concentration in the sample (mg/mL), *n* is the dilution factor of the extract, and *M* is the weight of the crude *D. pinnata* powder (g).

Exactly 10 mg of rutin reference substance was weighed, dissolved in MeOH, and volumetrically adjusted to 50 mL, yielding a 0.2 mg/mL stock solution. Aliquots ranging from 2 to 8 mL of the stock solution were transferred into separate 25 mL volumetric flasks and supplemented with distilled water to 8 mL. Afterwards, 1 mL of 5% sodium nitrite solution was added, and the mixture was left undisturbed for 6 min; then, 1.0 mL of 10% aluminum nitrate solution was added, with standing continued for another 6 min. Finally, 10 mL of 4% sodium hydroxide solution was added, and the volume was brought to the mark using distilled water. After complete mixing, the solution was maintained at room temperature for 15 min. With a reagent blank as control, a full-wavelength scan from 200 to 800 nm was conducted on a UV-Vis spectrophotometer, and 510 nm was determined as the maximum absorption wavelength. Absorbance values were recorded at this wavelength, and a calibration curve was plotted with concentration on the *X*-axis and absorbance on the *Y*-axis, resulting in the regression equation: *y* = 12.1163*x* − 0.0195520.

### 3.4. Single-Factor Experiments

Different types of NADES were screened, and the optimal solvent was determined using the flavonoid extraction rate as the index. Subsequently, the effects of the molar ratio of ChCl to urea (1:1, 1:2, 1:3, 1:4, 1:5, 2:1, 3:1), NADES water content (20%~70%), liquid-to-material ratio (20:1~60:1 mL:g), ultrasonic time (10~90 min), and ultrasonic temperature (30~70 °C) on the flavonoid extraction rate from *D. pinnata* were investigated [46].

### 3.5. Machine Learning Modeling

**Figure 12:**
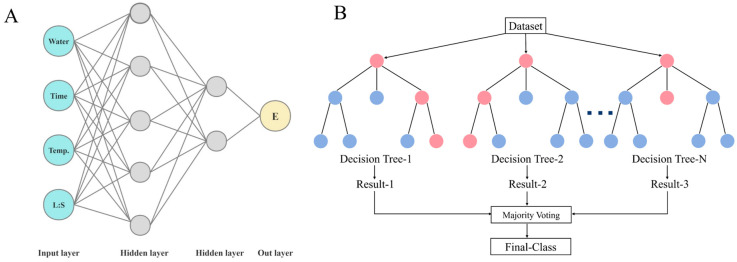
(**A**) Neural network structure; (**B**) random forest structure.

The experimental outcomes from 66 single-factor runs (including replicate runs) were processed with multilayer perceptron (MLP) and Random Forest (RF) algorithms implemented in Weka 3.8.6. The MLP model was structured with an input layer, hidden layer, and output layer (Figure 12A), taking ultrasonic temperature, extraction time, liquid-to-material ratio, and water content as input features, and flavonoid extraction rate as the response variable. To improve the robustness of the model evaluation with a small sample size, 10-fold cross-validation was applied, with 9 subsets for training and one for verification. Throughout model optimization, the hidden layer count and neuron number of MLP were tuned, and the parameter setting with the lowest RMSE and highest R was adopted to achieve superior prediction performance. The Random Forest model (Figure 12B) was also optimized using 10-fold cross-validation to optimize the number of decision trees and other parameters.

### 3.6. Response Surface Experiment

Based on the single-factor experiments, ultrasonic temperature (A), ultrasonic time (B), and liquid-to-material ratio (C) were identified as the key factors affecting flavonoid extraction efficiency. A three-factor, three-level Box–Behnken design was employed (Table 6), with flavonoid extraction yield as the response variable. The experimental data were analyzed, and the extraction procedure was optimized using Design-Expert 13.0. The second-order polynomial model of RSM was described in Equation (3).

(3) $$Y\left(X\right)=a_{0}+\sum_{i=1}^{n}a_{i}X_{i}+\sum_{i=1}^{n}b_{i}X_{i}^{2}+\sum_{i=1}^{n}\sum_{j=1}^{n}c_{\mathrm{ij}}X_{i}X_{j}$$

*Y* denotes the predicted response value; *a*_0_, *a_i_*, and *b_i_* represent the coefficients for the constant term, linear term, and quadratic term, respectively; *c_ij_* denotes the interaction coefficients of the influencing factors; and *n* denotes the number of influencing factors in the experiment.

### 3.7. Quantum Chemical Analysis of Flavonoids Extraction Mechanism

Quantum chemical calculations based on DFT were performed to explore the binding behaviors between rutin and different NADES solvents. Since rutin was employed as the standard reference in the experimental determination of flavonoid contents, it was also adopted as a typical flavonoid model for theoretical simulations. All molecular structures were optimized in Gaussian 16 at the B3LYP/6-31G(d,p) level [47]. Energy calculations were conducted at the M062X/6-31+G(d,p) level. The counterpoise correction was applied to eliminate the basis set superposition error (BSSE). The binding energy (ΔE) was calculated using Equation (4).

(4) $$\Delta E=E_{\mathrm{Com}\;}-\;(E_{\mathrm{Sol}}+E_{\mathrm{rutin}})+{\;E}_{\mathrm{BSSE}}$$

where *E_Com_* is the energy of the combination of solvent and rutin. *E_Sol_*, *E_rutin_*, and *E_BSSE_* are the energy of different solvents, rutin, and the basis set superposition error, respectively.

Visualization of intermolecular interactions was realized by electrostatic potential (ESP) and interaction region indicator (IRI) analyses using Multiwfn and VMD. The sign(λ_2_)ρ function was plotted on the IRI isosurface to classify the nature and strength of weak non-covalent interactions. In this expression, λ_2_ is the eigenvalue of the electron density Hessian matrix, and IRI was defined as Equation (5).

(5) $$\mathrm{IRI}(r)\;=|\nabla \rho (r)|/{[\rho (r)]}^{a}$$

*ρ* refers to the electronic density in quantum mechanics, *r* refers to the position vector of a specific point in space, and the parameter a in the IRI is set to 1.1.

### 3.8. Characterization

NADES and its constituent components were subjected to FTIR analysis using a Nicolet iS5 spectrometer (Thermo Fisher Scientific, Madison, WI, USA), with the scanning wavenumber ranging from 4000 to 500 cm^−1^ [48]. The extracted medicinal herb powder was dried and sputter-coated with gold, then observed under a SEM (Quanta 250, Thermo Fisher Scientific, USA) [49].

### 3.9. Antioxidant Activity

#### 3.9.1. Dpph· Radical Scavenging

Ethanol was employed to prepare a DPPH· radical working solution with OD = 1. The flavonoid extract and DPPH· solution were mixed at a volume ratio of 1:3, followed by incubation in the dark for 30 min. The absorbance A_1_ was measured at 520 nm using a microplate reader (Infinire M200pro, Tecan, Männedorf, Switzerland) [50]. Absorbance A_2_ was determined by replacing DPPH· with EtOH, while A_0_ was measured by replacing the sample with EtOH (Dai et al., 2024) [50]. The scavenging capacity against DPPH· radicals was computed on the basis of Equation (6).

(6) $$\mathrm{DPPH}\;\mathrm{Free}\;\mathrm{Radical}\;\mathrm{Scavenging}\;\mathrm{Rate}(\%)=\;(A_{0}-(A_{1}-A_{2}))\;/{\;A}_{0}\;\times \;100\%$$

#### 3.9.2. Abts^+^· Radical Scavenging

A stock solution of ABTS^+^· radicals was prepared and adjusted to an OD value of 0.8 using distilled water [51]. The flavonoid extract and ABTS^+^· reagent were mixed at a volume ratio of 1:3 and kept in the dark for 15 min, and the absorbance A_3_ was recorded at 734 nm. Absorbances A_4_ and A_5_ were determined using distilled water instead of ABTS^+^· and the sample, respectively. The scavenging activity against ABTS^+^· radicals was computed using Equation (7).

(7) $$\mathrm{ABTS}\;\mathrm{Free}\;\mathrm{Radical}\;\mathrm{Scavenging}\;\mathrm{Rate}\;(\%)=\;(A_{5}-(A_{3\;}-{\;A}_{4}))\;/\;A_{5}\;\times \;100\%$$

### 3.10. Antibacterial Activity

Antibacterial effects of the flavonoid extract against *S. aureus* and *E. coli* were assessed via the plate colony counting method [52]. The extract was mixed and diluted with bacterial suspension (OD_600_ = 0.5) at an appropriate ratio. A 30 μL volume of the sample was transferred and evenly coated onto agar plates, followed by incubation at 37 °C for 24 h before photographing and colony counting.

The flavonoid extract (50 μL) was taken and added to 200 μL of bacterial suspension with an OD_600_ value of 1. After incubating for 1 h, the mixture was subjected to centrifugation, and the sediment obtained was washed twice using physiological saline. The sample was fixed with 4% glutaraldehyde at 4 °C for 12 h, dehydrated in an EtOH gradient (30–100%), and resuspended in EtOH. A 10 μL aliquot was dropped onto a slide, followed by critical-point drying and gold sputtering. The bacterial ultrastructure was observed using a SEM (Hitachi, Regulus 8100, Japan) [53].

### 3.11. Data Analysis

Triplicate repetitions were performed for all experiments; data analysis and graph construction were conducted with the aid of software, including Origin 2024 and SPSS 27.0. The results were expressed as mean ± SD, and significant differences among groups (*p* < 0.05) were denoted by different letters.

## 4. Conclusions

This study developed a green and efficient NADES-UAE technology for extracting flavonoids from *D. pinnata*, with ChCl–urea (NADES-13) as the optimal solvent. The optimal parameters determined by RSM were as follows: ChCl–urea molar ratio of 1:3, water content of 60%, liquid-to-material ratio of 28.5 mL/g, ultrasonic treatment for 49 min, and temperature of 62 °C. Under these conditions, the extraction rate reached 117.95 ± 5.97 mg/g, which was 73.5% higher than that of 80% EtOH extraction. To further validate the reliability and universality of the extraction process, this study introduced two Machine Learning models, Neural Networks and Random Forests, and systematically compared them with RSM. The results show that RSM exhibits a correlation coefficient of R = 0.9981, a MAE of only 0.5373, and a RMSE of 0.6570, demonstrating superior fitting accuracy and prediction stability under small-sample conditions. Although Neural Networks (R = 0.9427, MAE = 4.3552, RMSE = 5.261) and Random Forests (R = 0.9431, MAE = 4.2762, RMSE = 5.2442) show some larger deviations, the overall trends are consistent with those of RSM, collectively confirming the intrinsic relationship between the extraction process parameters and flavonoid extraction rate. This provides multi-dimensional statistical support for process optimization. DFT analysis revealed the extraction mechanism: hydrogen bonds, Van der Waals forces, and cation–π interactions mediate the interaction between NADES-13 and flavonoids, leading to the formation of stable supramolecular structures. SEM and FTIR confirmed the ultrasonic cavitation effect and the hydrogen-bond network of NADES, respectively. In vitro experiments showed that the extract exhibited concentration-dependent antioxidant activity and strong antibacterial activity against *E. coli* and *S. aureus* under the tested conditions, with an inhibition rate of 96.87 ± 5.09% against *E. coli* at 0.04 mg/mL. The antibacterial mechanism was associated with the disruption of bacterial cell wall and membrane integrity. Overall, these results indicate that the optimized NADES-derived flavonoid extract has promising antibacterial potential against these two representative bacterial strains. This study is the first to apply NADES-UAE technology to this plant, innovatively integrating dual-algorithm optimization with quantum chemical mechanism analysis, and systematically validating its biological activities. It constructs a “Smart Optimization–Molecular Mechanism–Activity Verification” green extraction system, providing an efficient and environmentally friendly solution for the green extraction of plant bioactive components. This technology holds potential applications in the food, healthcare, and pharmaceutical industries, contributing to green chemistry and sustainable development. However, the regeneration and reusability of NADES-13 during the extraction of flavonoids from *D. pinnata* were not investigated in the present work, and future research should focus on solvent recovery, recycling efficiency, and its stability to support industrial applications.

## Figures and Tables

**Table 1:** Input feature weights and importance ranking.

Feature Parameter	Node1	Node2	Node3	Node4	Node5	Sum of Absolute Weights	Importance Rank
Water content	−2.649	−1.447	3.443	−9.475	−2.173	19.187	1
Ultrasonic time	−4.732	−4.643	−2.196	−3.501	−3.473	18.545	2
Ultrasonic temperature	−1.327	−4.687	−1.340	1.074	−2.176	10.604	3
Liquid-to-material ratio	3.555	−0.423	0.107	2.234	−1.538	7.857	4

**Table 2:** Feature ablation test and importance ranking.

Removed Feature	Correlation Coefficient R	Root-Mean-Square Error RMSE	ΔR (Baseline R = 0.943)	ΔRMSE (Baseline RMSE = 5.2613)	Importance Ranking
Water content	0.572	12.9894	−0.371	+7.7281	1
Ultrasonic time	0.7688	9.9940	−0.1742	+4.7327	2
Ultrasonic temperature	0.8460	8.3055	−0.097	+3.0442	3
Liquid-to-material ratio	0.8763	7.5483	−0.0667	+2.2870	4

**Table 3:** Results of the analysis of variance.

Source	Sum of Squares	Df	Mean Square	F-Value	*p*-Value	Significance
Model	1948.58	9	216.51	206.51	<0.0001	***
A—Ultrasonic temperature	68.98	1	68.98	65.79	<0.0001	***
B—Ultrasonic time	2.66	1	2.66	2.53	0.1555	-
C—liquid-to-material ratio	23.72	1	23.72	22.63	0.0021	**
AB	272.52	1	272.52	259.93	<0.0001	***
AC	0.22	1	0.22	0.21	0.6640	-
BC	22.11	1	22.11	21.09	0.0025	**
A^2^	218.23	1	218.23	208.15	<0.0001	***
B^2^	1064.12	1	1064.12	1014.97	<0.0001	***
C^2^	149.08	1	149.08	142.20	<0.0001	***
Residual	7.34	7	1.05			
Lack of fit	3.27	3	1.09	1.07	0.4549	-
Pure error	4.07	4	1.02			
Cor total	1955.92	16				
R^2^ = 0.9962, R_Adj_^2^ = 0.9914, R_Pre_^2^ = 0.9700						

** (*p* < 0.01) highly significant, and *** (*p* < 0.001) extremely significant.

**Table 4:** Comparison of models for three algorithms.

Metric	Neural Network	Random Forest	Response Surface
R	0.9427	0.9431	0.9981
MAE	4.3552	4.2762	0.5373
RMSE	5.261	5.2442	0.6570

**Table 5:** Formulation of NADES.

Solvent Abbreviation	Hydrogen Bond Donor (HBD)
NADES-1	Acetamide
NADES-2	Ethylene glycol
NADES-3	1,2-Propanediol
NADES-4	1,3-Propanediol
NADES-5	1,4-Butanediol
NADES-6	Lactic acid
NADES-7	Glycerol
NADES-8	Glucose
NADES-9	Acetic acid
NADES-10	Malic acid
NADES-11	Xylitol
NADES-12	Citric acid
NADES-13	Urea

Footnote: The hydrogen-bond acceptor (HBA) of all NADESs was choline chloride.

**Table 6:** Box–Behnken design factor level table.

Level	A: Ultrasonic Temperature (°C)	B: Ultrasonic Time (min)	C: Liquid-to-Material Ratio (mL:g)
−1	40	30	20
0	50	50	30
1	60	90	40

## Data Availability

The raw data supporting the conclusions of this article will be made available by the authors on request.
